# Academic BCMA CAR-T (ARI0002h) achieves MRD-negative remission with sBCMA biomarker response in relapsed POEMS syndrome after double ASCT

**DOI:** 10.1007/s00277-026-07063-4

**Published:** 2026-05-12

**Authors:** Víctor Torrecillas-Mayayo, Aina Oliver-Caldes, Nuria Martínez-Cibrian, Nil Albiol, Luis Gerardo Rodríguez-Lobato, Marta Español-Rego, Valentín Ortiz-Maldonado, Laura Rosiñol, Natalia Tovar, Sergio Navarro-Velasquez, Paola Charry, Daniel Munárriz, Jose Miguel Mateos, Carlos Jimenez-Mira, David F. Moreno, Mercedes Montoro, Pilar Ayora, Julio Delgado, Manel Juan, Azucena González-Navarro, Maria Teresa Cibeira, Carlos Fernández de Larrea

**Affiliations:** Hospital Clínic de Barcelona, IDIBAPS, Universitat de Barcelona, Barcelona, Spain

**Keywords:** POEMS syndrome, CAR-T, BCMA

## Abstract

Polyneuropathy, Organomegaly, Endocrinopathy, Monoclonal gammopathy, and Skin changes (POEMS) syndrome is a rare plasma cell disorder with diverse multisystem manifestations. Autologous stem cell transplantation (ASCT) is the preferred front-line therapy when feasible, but relapsed/refractory (RR) cases after ASCT failure lack effective salvage options. B-cell maturation antigen (BCMA)-directed CAR-T cell therapy has revolutionised multiple myeloma (MM) treatment. Given the shared BCMA expression on clonal plasma cells between MM and POEMS, CAR-T cell therapy is also a biologically compelling strategy for POEMS syndrome [1]. However, there are only two previous case reports of CAR-T cell therapy in POEMS [3,4], none involving patients with prior ASCT failure. We report a 54-year-old male with RR-POEMS progressing after two previous ASCT failures who received an academic point-of-care BCMA-directed CAR-T cell therapy (ARI0002h) via fractionated infusion. No cytokine release syndrome (CRS) or neurotoxicity (ICANS) occurred. At 3 months, the patient achieved a measurable residual disease (MRD)-negative complete haematological response (CR_H_) by Next Generation Flow (NGF) with a sensitivity of 10⁻⁶ and a radiological CR. Responses have been sustained for 10 months. Serum BCMA (sBCMA) levels declined dynamically in parallel with haematological response, suggesting potential utility as a novel monitoring biomarker in POEMS. This case supports BCMA-directed CAR-T therapy as a safe and effective salvage strategy in RR-POEMS following ASCT failure, even in an aggressive disease context.

Clinical trial registration: Not aplicable.

## Introduction

Polyneuropathy, Organomegaly, Endocrinopathy, Monoclonal gammopathy, and Skin changes (POEMS) syndrome is a rare plasma cell disorder with an estimated prevalence of approximately 1–3 cases per million [1]. It is characterized by the overproduction of vascular endothelial growth factor (VEGF) and pro-inflammatory cytokines, resulting in multisystem manifestations. Given its low prevalence, conducting prospective studies to validate optimal treatment strategies is extremely difficult, and therapeutic decisions are largely extrapolated from multiple myeloma (MM), a more common plasma cell dyscrasia sharing pathophysiology and cell of origin [1].

Autologous stem cell transplantation (ASCT) is the preferred front-line therapy for transplant-eligible patients, with reported hematological complete response (CR_H_) rates of 29–57% [2]. However, ASCT is not curative, and POEMS syndrome frequently relapses [1]. No prospective data exist to guide treatment decisions in relapsed/refractory (RR) POEMS after ASCT failure, and reported strategies –including lenalidomide, daratumumab, or repeat ASCT– remain largely exploratory and are often ineffective [2].

B-cell maturation antigen (BCMA)-directed CAR-T cell therapy has revolutionized the treatment of RR-MM. Given the shared BCMA expression on clonal plasma cells in both MM and POEMS syndrome, CAR-T cell therapy represents a biologically compelling strategy for RR-POEMS [2]. However, experience remains extremely limited, with only two case reports of CAR-T cell therapy in POEMS syndrome published to date, and neither involved patients relapsing after ASCT [3, 4].

ARI0002h is an academic humanized 4-1BB CAR-T targeting BCMA tested in the CARTBCMA-HCB-01 clinical trial for RR-MM patients, showing overall and CR rates of 95% and 58%, respectively [5, 6]. ARI0002h CAR-T is infused in a fractionated manner, with three initial aliquots containing respectively 10, 30, and 60% of the total CAR-T cell product, followed by a booster dose administered at least 3 months after the initial infusion in patients with any positive response and no limiting toxicities. The rationale behind the booster dose is to consolidate and deepen responses, and to re-expand CAR-T cells in the peripheral blood that may be decaying or entering an exhausted state [5, 6].

Here, we report the third case worldwide of CAR-T cell therapy in POEMS syndrome and the first using a point-of-care manufactured academic CAR-T as ARI0002h. Notably, this is the first report of a patient achieving a measurable residual disease (MRD)-negative CR after failing two ASCTs, demonstrating the safety and efficacy of CAR-T in highly challenging RR-POEMS scenarios, with a sustained response at 10 months of follow-up. We also provide insight into the use of serum BCMA (sBCMA) as a novel biomarker in POEMS to monitor treatment response.

## Case report

A 36-year-old male presented to our hospital in 2007 with complaints of hypoesthesia and weakness in both feet, having been followed for an IgA lambda monoclonal gammopathy of uncertain significance for 3 years. After a thorough assessment, he fulfilled POEMS diagnostic criteria [1], presenting with polyneuropathy and a monoclonal plasma-cell disorder, with 7% lambda-restricted plasma cells in bone marrow aspirate (BMA) and detection of an IgA-lambda M-protein of 7 g/L with hypogammaglobulinemia. Additional criteria included sclerotic bone lesions in the spine, pelvis, femurs, and tibias; central hypogonadism; multiple angiomas; and thrombocytosis. First-line therapy with ASCT using melphalan 200 mg/m^2^ achieved a very good partial hematological response (VGPR_H_) with persistent IgA-lambda immunofixation positivity and clinical improvement. The first determination of VEGF in this patient was performed two years after ASCT and showed levels within the normal range (< 16 pg/mL).

Nine years after ASCT, the patient developed worsening polyneuropathy, new skin lesions (angiomas, xerosis, and Raynaud phenomenon), extensive sclerotic lesions, bilateral papilledema, and extravascular volume overload with ascites, severe edema in the lower extremities, and bilateral pleural effusion. M-protein was 2 g/L, and VEGF levels increased to 310 pg/ml. Two cycles of cyclophosphamide, bortezomib, and dexamethasone (CyBorD regimen) produced no response. A second ASCT was then performed. The patient developed a severe engraftment syndrome requiring intensive care management and high-dose corticosteroids. Upon recovery, the patient achieved VGPR_H_ and a clinical response with improvement in polyneuropathy symptoms and resolution of papilledema and extravascular decompensation.

**Fig. 1:**
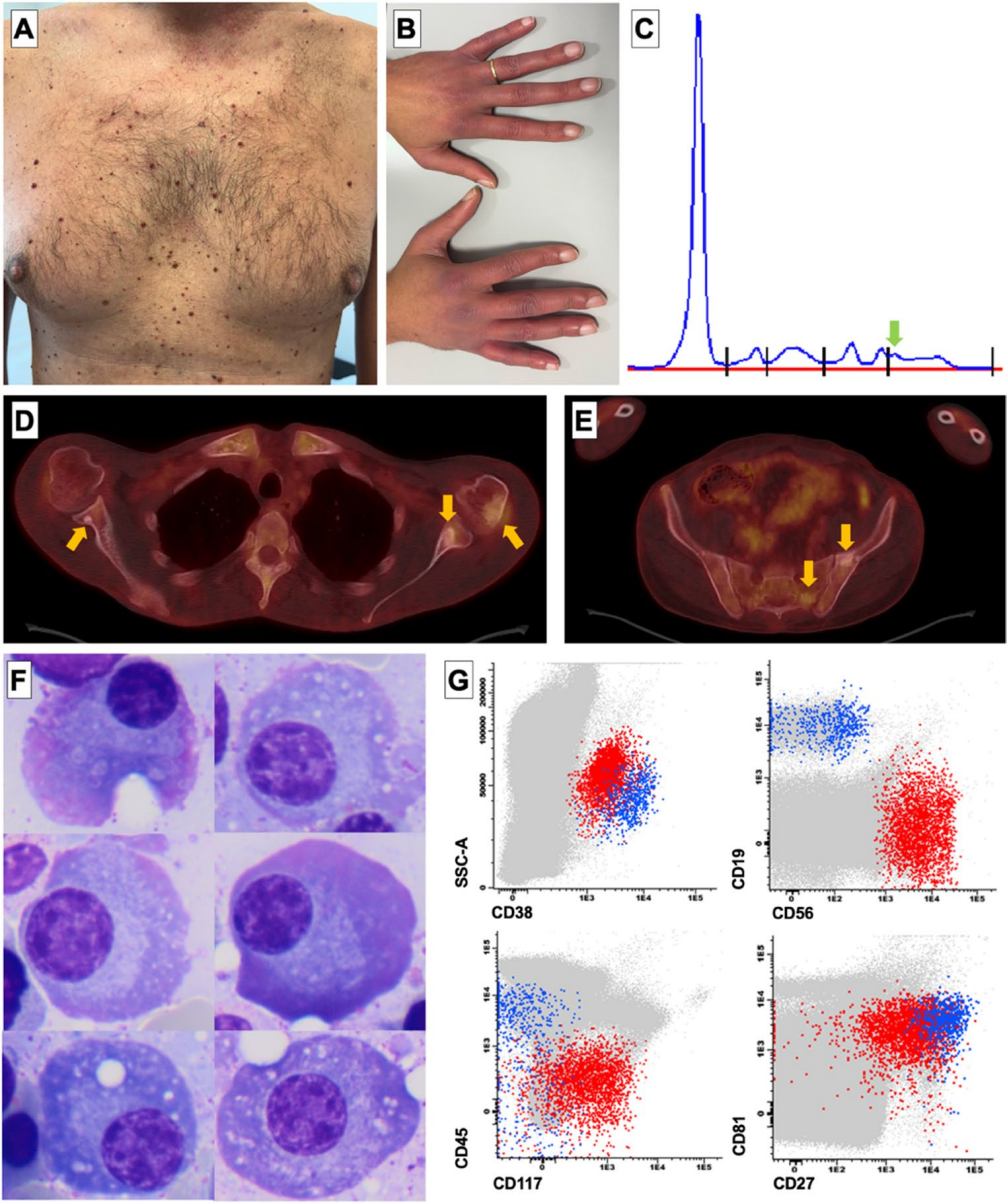
Patient findings before treatment with ARI0002h. (A): Cutaneous angiomas. (B): Raynaud syndrome. (C): Monoclonal IgA-lambda migrating in the gamma region on serum electrophoresis (arrow). (D-E): PET-CT showing sclerotic lesions in multiple bones, including humeri, scapulae, iliac pale, and sacrum (arrow), some of them with FDG-18 uptake. (F): Bone marrow aspirate assessment at 100x magnification, showing multiple aberrant plasma cells, some of them with flame cell morphology. (G): Plasma cells immunophenotype comparing normal plasma cells (blue) and aberrant plasma cells (red). Aberrant plasma cells showed CD38^dim^, CD45-, CD19-, CD56+, CD117^dim^, CD81+

**Fig. 2:**
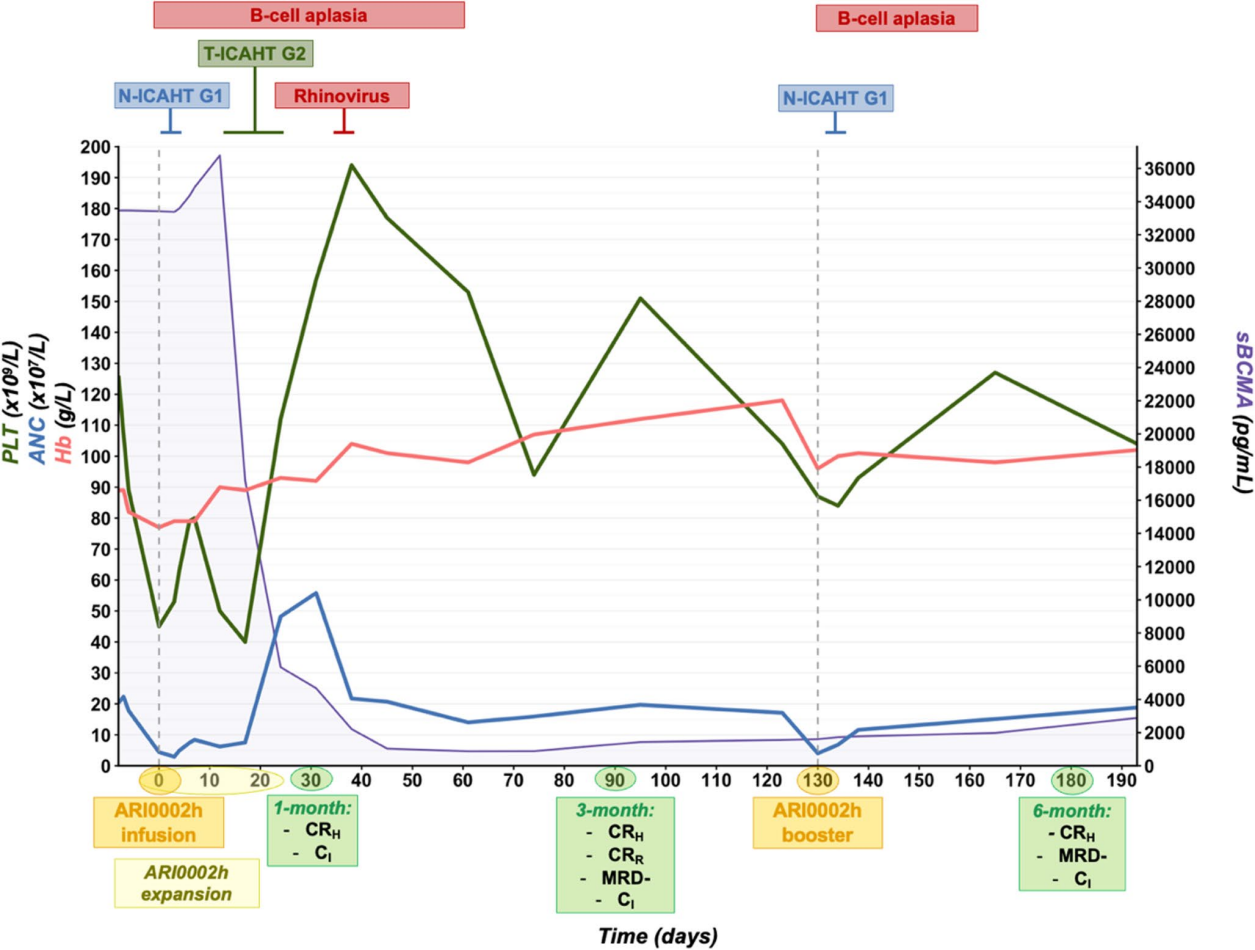
Clinical outcomes, blood counts and sBCMA kinetics after ARI0002h infusion. ANC: Absolute neutrophil count; CR_H_: Hematological complete response; C_I_: Clinical improvement; CR_R_: Radiological complete response; G: Grade; Hb: Hemoglobin; N-ICAHT and T-ICATH: Neutrophil and thrombocytopenia immune effector cell-associated hematotoxicity; MRD-: Negative measurable residual disease; PLT: Platelet count; sBCMA: Serum B-cell maturation antigen

Eight years after the second ASCT, progressive disease reemerged with lower-extremity edema, Raynaud phenomenon, and anemia (hemoglobin 90 g/l). M-protein was 2 g/L, and BMA showed 20% lambda-restricted plasma cells with an aberrant immunophenotype and normal cytogenetics. PET-CT revealed multiple new sclerotic lesions, some with FDG-18 uptake. Findings are shown in Fig. 1. At this moment, the patient was 54 years old, and given the aggressive course of the previous relapse, ARI0002h CAR-T was offered on a compassionate-use basis. Baseline CAR-HEMATOTOX score [7] was 1, EASIX score [8] 0.43, and M-EASIX [9] 0.49. Interestingly, VEGF levels at this moment were within range (85 pg/mL), but sBCMA levels were substantially high (33,500 pg/mL). Therefore, sBCMA levels were used as a biomarker for CAR-T response. After lymphodepletion (LD) with fludarabine (total dose 90 mg/m^2^) and cyclophosphamide (total dose 900 mg/m^2^), an infusion of 3 × 10^6^ ARI0002h cells/kg was administered in a fractionated manner: 10% on day 0, 30% on day + 3, and 60% on day + 7. Vein-to-vein time was 31 days. Blood counts and sBCMA dynamics are shown in Fig. 2.

No cytokine release syndrome (CRS) or immune effector cell-associated neurotoxicity syndrome (ICANS) occurred. Early immune effector cell-associated hematotoxicity (ICAHT), characterized by neutropenia-ICAHT (N-ICAHT) grade 1 [10] and thrombocytopenia-ICAHT (T-ICAHT) grade 2 [11], was observed. B-cell aplasia lasted 2 months. Pre-existing hypogammaglobulinemia persisted, and monthly immunoglobulin replacement was initiated. Infectious prophylaxis consisted of cotrimoxazole and acyclovir. Cefixime, fluconazole, and weekly G-CSF were added during neutropenic periods. A rhinovirus infection 1 month after infusion resolved without treatment.

At the 1-month assessment, the patient achieved CR_H_, with edema resolution, increased hemoglobin levels, and stable cutaneous lesions. CAR-T cell expansion was detected in peripheral blood until day + 22, coinciding with the peak of expansion. At the 3-month assessment, the patient continued in CR_H_ and was asymptomatic. MRD-negative disease was confirmed in BMA by Next-Generation Flow (NGF) with a sensitivity of 10^− 6^. PET-CT showed radiological CR, and sBCMA decreased to 1450 pg/mL. VEGF persisted within normal range (120 pg/mL).

A non-fractionated booster dose of 3 × 10^6^ ARI0002h cells/kg was administered on day + 130 within the specialized at-home care program. Per protocol, a new LD with fludarabine and cyclophosphamide was repeated before the booster since CAR-T cell persistence was no longer detected in peripheral blood [5, 6]. No CRS, ICANS, or infectious complications were observed. An early N-ICAHT grade 1, managed with weekly G-CSF for 2 weeks, was the only complication reported. B-cell aplasia recovered within 1 month.

Although in vivo CAR-T cell expansion in peripheral blood could not be detected after the booster dose, at the 6-month assessment, the patient persisted in CR_H_, with normal-range VEGF levels (85 pg/mL), and MRD-negative (sensitivity 10^− 6^). Ten months after infusion, the patient remains in CR_H_, with the next comprehensive assessment planned at 12 months.

## Discussion

We present a patient with RR-POEMS who was treated with ARI0002h academic BCMA CAR-T cell therapy. The phenotype of this patient’s disease was aggressive and atypical, showing unusually high plasma cell burden, which we attribute to clonal selection under pressure from multiple prior lines of therapy, and with anemia probably secondary to plasma cell infiltration in the bone marrow. Although unusual, this presentation does not invalidate the POEMS diagnosis since the cardinal findings in this patient were polyneuropathy, volume overload, progressive sclerotic bone lesions, and skin alterations. Precisely, this type of aggressive RR-POEMS disease reinforces the unmet need for intensive and effective salvage options for these patients.

We provide data showing that CAR-T cell therapy was well tolerated, safe, and effective in this patient despite the previously discussed aggressive phenotype, achieving the deepest response described in the literature in POEMS after CAR-T cell therapy (MRD-negative CR by NGF with 10^− 6^ sensitivity; Table 1). Interestingly, the responses achieved with ARI0002h in our patient were deeper than those previously obtained with prior ASCTs (VGPR_H_). Although the short follow-up of our patient (10 months) represents an important limitation of this report, it is well established that deeper responses in POEMS correlate with longer progression-free survival and overall survival [12, 13]; the scarce evidence of efficacy and tolerability of CAR-T cell therapy in POEMS proves this case to be a valuable example of how BCMA-directed CAR-T cell therapy could be an effective salvage option after ASCT in RR-POEMS.

Notably, our patient did not experience CRS or ICANS despite a high baseline tumor burden and a history of severe engraftment syndrome. This supports the safety benefit of the fractionated infusion, which was already observed in the CARTBCMA-HCB-01 trial [5, 6]. Interestingly, our patient was already in CR at the time of the booster dose, as did the 45% of patients in the original cohort [5]. It remains unknown whether these patients benefit from the booster strategy in terms of duration of response, as the original study lacked a control arm without a booster [5]. At present, all patients undergoing ARI0002h CAR-T who are eligible continue to receive the booster dose.

**Table 1:** Reported cases of CAR-T therapy in POEMS syndrome

	Xu et al. (2018)	Williams et al. (2025)	Present case (2026)
Age/Sex	49/female	73/male	54/male
Previous lines of treatment	Len-D	Dara-Len-D and Dara-Pom-D	2 ASCT and CyBorD
Previous ASCT	No (rejected by patient)	No	Yes (2 previous ASCT)
CAR-T	Murine anti-BCMA	Cilta-cel (commercial)	ARI0002h (academic)
Type of use	Phase I clinical trial for MM	Compassionate	Compassionate
Dose	1 × 10^7^/kg	0.6 × 10^8^ total cells	3 × 10^6^/kg fractionated (10/30/60%) + 3 × 10^6^/kg booster
Lymphodepletion	Flu + Cy	Flu + Cy	Flu + Cy
CRS	Grade 1	Grade 3	None
ICANS	None	None	None
ICAHT	Not reported	Not reported	N-ICAHT grade 1 and T-ICAHT grade 2
Best response	sCR	sCR	sCR with MRD-negative (10^− 6^ sensitivity)
PET-CT assessment	Not reported	Radiological CR at 1 month	Radiological CR at 3 months
Baseline % plasma cells	3%	20%	20%
Baseline sBCMA	1,000 pg/mL	60 pg/mL	33,500 pg/mL
Post CAR-T sBCMA	0 pg/mL (100% decrease)	Approximately 8 pg/mL (87% decrease)	1,450 pg/mL (96% decrease)
Follow-up	10 months on response	6 months on response	10 months on response

*ASCT* Autologous stem cell transplantation, *BCMA* B-cell maturation antigen, *CyBorD* Cyclophosphamide + Bortezomib +Dexamethasone, *CR* Complete response, *D* Dexamethasone, *Dara* Daratumumab, *Flu* Fludarabine, *ICAHT* Immune effector cell-associated hematotoxicity, *ICANS* Immune effector cell-associated neurotoxicity syndrome, *Len* Lenalidomide, *MM* Multiple myeloma, *MRD* Minimal residual disease, *N-ICAHT* Neutropenia-ICAHT, *Pom* Pomalidomide, *sBCMA* Soluble B-cell maturation antigen, *sCR* Stringent complete response, *T-ICAHT* Thrombocytopenia-ICAHT

Beyond clinical and radiological responses, biomarker monitoring provided additional insight into disease dynamics in our patient. It is well recognised that VEGF is an imperfect marker, since discordance between disease activity and VEGF response has been documented, and clinical decisions should be guided by trends rather than absolute values [1]. As discussed, our patient presented normal VEGF levels before infusion of ARI0002h, but high sBCMA levels that were consequently monitored as a response marker. sBCMA is shed from the surface of clonal plasma cells and has demonstrated prognostic value as a tumor marker in MM [14], smoldering MM [15], and monoclonal gammopathies of undetermined significance [15], with higher baseline levels correlating with worse clinical outcomes and greater CRS severity in patients treated with ARI0002h in the CARTBCMA-HCB-01 trial [6]. However, its role in POEMS syndrome has not been clearly established, and prior reports have not consistently demonstrated significant sBCMA elevation in this disease [14].

The biological basis for sBCMA heterogeneity observed across POEMS cases warrants further discussion. As a general principle, higher plasma cell burden correlates with higher sBCMA levels. However, studies performed mainly in MM patients showed that sBCMA levels also reflect the complex interaction between membrane BCMA density [16], γ-secretase activity –the enzyme responsible for shedding membrane BCMA into sBCMA– [17], BCMA glycosylation state [18], and APRIL and BAFF cytokines production by the tumor microenvironment [19]. Significant sBCMA heterogeneity has been reported between patients with similar plasma cell burden, but also between subclones within the same tumor [20]. This could explain why our case and the previous POEMS syndrome case reports treated with CAR-T cell therapy did not present a linear correlation between bone marrow plasma cells and sBCMA levels [3, 4]. Despite these differences in baseline levels, all three patients experienced a marked decrease in sBCMA levels paralleling hematological response [3, 4]. To our knowledge, regulatory sBCMA mechanisms and sBCMA prognostic value as a biomarker have not been extensively studied in POEMS syndrome, and we highlight this as an important gap for future research.

## Conclusion

Overall, this case suggests that BCMA-directed CAR-T cell therapy represents an effective and well-tolerated salvage therapy in RR-POEMS after ASCT failure. Specifically, it demonstrates that deep MRD-negative complete responses are achievable in this setting, that the fractionated infusion of ARI0002h confers a favorable safety profile, and that sBCMA may serve as a dynamic response biomarker in the absence of reliably elevated VEGF. However, longer follow-up and larger POEMS cohorts treated with CAR-T cell therapy are needed to confirm these findings. We also highlight the gap in the literature regarding sBCMA regulatory mechanisms and prognostic potential in the specific context of POEMS syndrome.

## Data Availability

To protect patient confidentiality, the individual patient data underlying this case report cannot be shared publicly. Any inquiries regarding the case may be directed to the corresponding author, who will be pleased to respond to reasonable requests.

## Declarations

### Permission to reproduce material from other sources

Not aplicable.

## References

[1] Dispenzieri A (2023) POEMS syndrome: Update on diagnosis, risk-stratification, and management. Am J Hematol 98:1934–1950. 10.1002/ajh.27081 37732822 10.1002/ajh.27081

[2] Khwaja J, D’Sa S, Lunn MP, Sive J (2023) Evidence-based medical treatment of POEMS syndrome. Br J Haematol 200:128–136. 10.1111/bjh.18400 35934319 10.1111/bjh.18400

[3] Xu J, Wang Q, Xu H et al (2018) Anti-BCMA CAR-T cells for treatment of plasma cell dyscrasia: case report on POEMS syndrome and multiple myeloma. J Hematol Oncol 11:128. 10.1186/s13045-018-0672-7 30348186 10.1186/s13045-018-0672-7 PMC6198365

[4] Williams M, Atanackovic D, Mulatu R et al (2025) Cilta-cel CAR T cells as an effective and well tolerated treatment for POEMS: a case study. Haematologica. 10.3324/haematol.2025.287702 40501407 10.3324/haematol.2025.287702 PMC12580721

[5] Oliver-Caldés A, González-Calle V, Cabañas V et al (2023) Fractionated initial infusion and booster dose of ARI0002h, a humanised, BCMA-directed CAR T-cell therapy, for patients with relapsed or refractory multiple myeloma (CARTBCMA-HCB-01): a single-arm, multicentre, academic pilot study. Lancet Oncol 24:913–924. 10.1016/S1470-2045(23)00222-X 37414060 10.1016/S1470-2045(23)00222-X

[6] Oliver-Caldes A, Español-Rego M, Zabaleta A et al (2024) Biomarkers of Efficacy and Safety of the Academic BCMA-CART ARI0002h for the Treatment of Refractory Multiple Myeloma. Clin Cancer Res 30:2085–2096. 10.1158/1078-0432.CCR-23-3759 38466644 10.1158/1078-0432.CCR-23-3759

[7] Rejeski K, Hansen DK, Bansal R et al (2023) The CAR-HEMATOTOX score as a prognostic model of toxicity and response in patients receiving BCMA-directed CAR-T for relapsed/refractory multiple myeloma. J Hematol Oncol 16:88. 10.1186/s13045-023-01465-x 37525244 10.1186/s13045-023-01465-x PMC10391746

[8] Frenking JH, Zhou X, Wagner V et al (2024) EASIX-guided risk stratification for complications and outcome after CAR T-cell therapy with ide-cel in relapsed/refractory multiple myeloma. J Immunother Cancer 12:e009220. 10.1136/jitc-2024-009220 39379098 10.1136/jitc-2024-009220 PMC11459298

[9] Moreno-Castaño AB, Fernández S, Brillembourg H et al (2026) m-EASIX (better than EASIX) predicts severe CAR T-cell toxicities, worse overall survival, and discriminates cytokine release syndrome from sepsis. Front Immunol 16. 10.3389/fimmu.2025.1664788 10.3389/fimmu.2025.1664788 PMC12819699 41573561

[10] Rejeski K, Subklewe M, Aljurf M et al (2023) Immune effector cell-associated hematotoxicity: EHA/EBMT consensus grading and best practice recommendations. Blood 142:865–877. 10.1182/blood.2023020578 37300386 10.1182/blood.2023020578

[11] Rejeski K, Sanz J, Fei T et al (2025) T-ICAHT: grading and prognostic impact of thrombocytopenia after CAR T-cell therapy. Blood 146:834–846. 10.1182/blood.2025028833 40258181 10.1182/blood.2025028833 PMC12783514

[12] Li A, Gao X, Zhao H et al (2024) Long-Term Outcomes of Autologous Stem Cell Transplantation in Patients with Newly Diagnosed POEMS Syndrome. Transplantation Cell Therapy 30. 10.1016/j.jtct.2023.11.001. :207.e1-207.e7 10.1016/j.jtct.2023.11.001 37931801

[13] Fang F, Lan X-X, Hu R-H et al (2024) Efficacy of bortezomib, cyclophosphamide, and dexamethasone for newly diagnosed POEMS syndrome patients. Ther Adv Neurol Disord 17:17562864231219151. 10.1177/17562864231219151 38288324 10.1177/17562864231219151 PMC10823847

[14] Guo P, Wang Y, He H et al (2024) Elevated serum levels of soluble B-cell maturation antigen as a prognostic biomarker for multiple myeloma. Clin Exp Immunol 217:221–232. 10.1093/cei/uxae043 38743453 10.1093/cei/uxae043 PMC11310710

[15] Visram A, Soof C, Rajkumar SV et al (2021) Serum BCMA levels predict outcomes in MGUS and smoldering myeloma patients. Blood Cancer J 11:120. 10.1038/s41408-021-00505-4 34168119 10.1038/s41408-021-00505-4 PMC8225625

[16] Zhang M, Gray F, Cushman I et al (2023) A Novel BCMA Immunohistochemistry Assay Reveals a Heterogenous and Dynamic BCMA Expression Profile in Multiple Myeloma. Mod Pathol 36:100050. 10.1016/j.modpat.2022.100050 36788077 10.1016/j.modpat.2022.100050

[17] Laurent SA, Hoffmann FS, Kuhn P-H et al (2015) γ-Secretase directly sheds the survival receptor BCMA from plasma cells. Nat Commun 6:7333. 10.1038/ncomms8333 26065893 10.1038/ncomms8333 PMC4490565

[18] Huang H-W, Chen C-C, Lin K-I et al (2024) Single Site N-Glycosylation of B Cell Maturation Antigen (BCMA) Inhibits γ-Secretase-Mediated Shedding and Improves Surface Retention and Cell Survival. ACS Chem Biol 19:153–161. 10.1021/acschembio.3c00592 38085681 10.1021/acschembio.3c00592

[19] Tai Y-T, Acharya C, An G et al (2016) APRIL and BCMA promote human multiple myeloma growth and immunosuppression in the bone marrow microenvironment. Blood 127:3225–3236. 10.1182/blood-2016-01-691162 27127303 10.1182/blood-2016-01-691162 PMC4920023

[20] Lee H, Ahn S, Maity R et al (2023) Mechanisms of antigen escape from BCMA- or GPRC5D-targeted immunotherapies in multiple myeloma. Nat Med 29:2295–2306. 10.1038/s41591-023-02491-5 37653344 10.1038/s41591-023-02491-5 PMC10504087

